# Primary lymphoma of the female genital tract masquerading as gynecological malignancy

**DOI:** 10.1186/s12905-024-03037-8

**Published:** 2024-04-18

**Authors:** Suhua Shi, Wuan Li, Guantai Ni, Jin Ding, Yinhua Liu, Haixing Wu, Zhen Zhang, Zhimin Ding

**Affiliations:** 1 Department of Obstetrics and Gynecology, The First Affiliated Hospital of Wannan Medical College, Wuhu, 241000 China; 2 Department of Pathology, The First Affiliated Hospital of Wannan Medical College, Wuhu, China; 3 Department of Radiology, The First Affiliated Hospital of Wannan Medical College, No. 2 Zheshan West Road, Wuhu, 241000 China

**Keywords:** Lymphoma, Female genital tract, Gynecological malignancy, B-cell lymphoma, Chemotherapy

## Abstract

**Background:**

Primary lymphoma of the female genital tract (PLFGT) is a rare malignant tumor in the female reproductive system, with a low incidence and few clinical reports. The aim of this study is to report our institutional experience with this rare malignancy and emphasize the need for increasing the awareness about PLFGT presenting with gynecologic symptoms.

**Methods:**

The medical records of patients diagnosed with PLFGT from March 2014 to November 2022 in the First Affiliated Hospital of Wannan Medical College were reviewed. Histological classification and staging were based on the World Health Organization and Ann Arbor systems, respectively.

**Results:**

There were 13 patients with diagnosis of PLFGT and the median length of follow-up was 31 months (0-102 months). The main clinical symptoms included postmenopausal vaginal bleeding, pelvic mass and abdominal pain. Serum LDH increased in 10 patients and serum CA125 elevated in 2 patients. The tumor of ovarian or uterine presented as solid masses in CT or MRI, and ascites was rare. The histological subtypes were diffuse large B-cell (*n* = 12) and follicular (*n* = 1) lymphoma. Tumors were located in ovary (*n* = 8), uterus (*n* = 3), and cervix (*n* = 2). According to the Ann Arbor staging system, 6 cases were classified as stage II and 7 cases were classified as stage IV, respectively. A total of 10 patients underwent surgery. Combination chemotherapy was used in 10 patients. Eight patients had tumor-free survival, 1 patient had recurrent disease, 3 patients died and 1 patient lost to follow-up. The median survival time was 32 months (1-102 months).

**Conclusion:**

PLFGT usually presents as gynecological symptoms and solid masses in pelvis. Surgery or biopsy was the way to obtain the pathologic diagnosis, and combination chemotherapy is the efficient method for PLFGT. Making an accurate preoperative diagnosis is of paramount importance to avoid radical gynecologic surgery.

## Background

Primary non-Hodgkin lymphoma (NHL) is a common hematological malignancies. It can arise from lymph nodes (nodal) or lymphatic cells located in solid organs (extranodal) [1]. Extranodal lymphoma accounts for about 25–35% of all NHL cases. It is considered to be a disease with unique clinical characteristics, epidemiological characteristics and prognosis [1–4]. Primary extranodal NHL most commonly arise in the gastrointestinal tract (30–40%), followed by the skin (10%) and the central nervous system (2–4%) [5]. Primary lymphoma of female genital tract((PLFGT) is an extremely rare entity, representing 0.2–1.1% of extra-nodal NHL lymphomas [6]. The most common histological subtype of PLFGT is diffuse large B-cell lymphoma [7]. And it often occurrs in the ovary, and cervix. Uterus corpus, vagina, and vulva are less common primary positions [8]. Patients with PLFGT in early stages are often asymptomatic, and common symptoms of more advanced stages are vaginal bleeding or discharge, abdominal pain, distension, and pelvic mass [9, 10]. In clinical practice, patients with PLFGT initially present to their gynecologists. This often leads to misdiagnosed and considered as primary genital malignancies, which delays the diagnosis and causes unappropriate clinical management. Therefore, it is important to establish its histological diagnosis as soon as possible.

Because of the rarity of this disease, there is no standard treatment for women with PFLGT. Most series describe that chemotherapy, either alone or in combination with surgery and radiotherapy is the mainstay of treatment for women with PFLGT. Cyclophosphamide, hydroxydaunorubicin, oncovin, and prednisone with rituximab (R-CHOP) is the most commonly used chemotherapy regimen [8]. Since both prognosis and treatment completely differ from other gynecological malignancies, interdisciplinary collaboration among gynecologists, radiologists, pathologists, and hematologists is very important.

In this paper, we report 13 patients presenting with PLFGT in the last 8 years of our hospital, and retrospectively analyze the clinical characteristics, diagnosis, treatment and prognosis. We expect our results can be helpful to gynecologists for improving the clinical diagnosis and treatment of PFLGT.

## Methods

A retrospective chart review was performed on patients with diagnosis of PLFGT from March 2014 to November 2022 in the First Affiliated Hospital of Wannan Medical College. The histological classification and diagnosis was based on the 2008 World Health Organization (WHO), and clinical staging was performed according to the Lymphoma Ann-Arbor (Cotswolds Revision) staging system. The patient’s age, clinical symptoms, location of lesion, staging, treatments, and clinical follow-up were recorded. The medical records of diagnostic imaging, levels of serum CA125 and lactate dehydrogenase (LDH), and pathology reports were reviewed.

All pathology specimens were obtained from patients hospitalized in the gynecological ward of our hospital. All pathology reports were reviewed by an on-site pathologist. The clinical follow-up data were collected from detailed review of all medical records and telephone interviews. The final follow-up was performed in August 2023. This study was approved by the Ethics Committee of the First Affiliated Hospital of Wannan Medical College. Informed consent was obtained from all patients or family members.

## Results

### Patients’ characteristics

We identified 13 patients with diagnosis of PLFGT. Patients ranged in age from 30 to 78 years, with a median age of 67 years at diagnosis. The main clinical symptoms included postmenopausal vaginal bleeding (*n* = 6), pelvic mass (*n* = 8), lower abdominal pain (*n* = 3), abdominal distension (*n* = 3) and vaginal discharge (*n* = 2). Among them, 10 patients were menopausal, and 3 patients had normal menstrual cycle. The complications included hypertension, diabetes, and coronary heart disease (CHD). Preoperative diagnosis was considered as ovarian cancers in 8 patients, uterine malignant tumors in 3 patients, cervical cancer in 2 patient (Table 1) .

**Table 1:** Clinical characteristics and treatment of 13 patients with PLFGT

Patient number	Age (year)	Clinical feature	Location	Size of lesion (Ultrasonograph, cm)	LDH (Normal:135-225u/L)	CA125 (Normal:0-35u/mL)	Surgery/biopsy
Case1	70	pelvic mass, postmenopausal vaginal bleeding	Bilateral ovaries	9.7	821	46.3	cytoreductive surgery (hysterectomy, bilateral adnexectomy and omentectomy)
Case2*	78	postmenopausal vaginal bleeding	Uterus	5.1	285	20.0	Diagnostic curettage
Case3**	58	postmenopausal vaginal bleeding	Cervix	4.3	121	6.7	Cervical puncture biopsy
Case4	33	pelvic mass lower abdominal pain,	Bilateral ovaries	15.0	1501	39	total hysterectomy, bilateral adnexectomy, pelvic and para-aortic lymph node dissection, omentectomy
Case5	67	pelvic mass, abdominal distension	Bilateral ovaries	5.8	180	25.2	total hysterectomy, bilateral adnexectomy, pelvic and para-aortic lymph node dissection, omentectomy
Case6	73	pelvic mass	Left ovary	4.5	430	30	Left adnexectomy and lymphatic biopsy
Case7	31	pelvic mass	Bilateral ovaries	14.4	284	24.4	cytoreductive surgery (hysterectomy, bilateral adnexectomy and omentectomy)
Case8	53	postmenopausal vaginal bleeding	Uterus	6.8	680	30.1	Diagnostic curettage
Case9^***^	70	postmenopausal vaginal bleeding, vaginal discharge	Uterus	8.9	296	17.1	total hysterectomy, bilateral adnexectomy, pelvic and para-aortic lymph node dissection, omentectomy
Case10	30	pelvic mass, lower abdominal pain, abdominal distension	Bilateral ovaries	8.7	2705	959.3	total hysterectomy and bilateral adnexectomy
Case11*	58	pelvic mass, lower abdominal pain	Right ovary	8.1	277	15.2	total hysterectomy and bilateral adnexectomy
Case12	72	pelvic mass, lower abdominal pain, abdominal distension	Bilateral ovaries	4.5	301	24.0	bilateral adnexectomy and lymphatic biopsy
Case13	77	postmenopausal vaginal bleeding, vaginal discharge	Cervix	3.5	382	31.5	Radical total hysterectomy, bilateral adnexectomy and pelvic lymph node dissection

* Complications of hypertension; ** Complications of diabetes; ***Complications of hypertension and coronary heart disease; PLFGT Primary lymphoma of the female genital tract;

LDH lactate dehydrogenase; CA125 carbohydrate antigen

### Characteristics of imaging

**Fig. 1:**
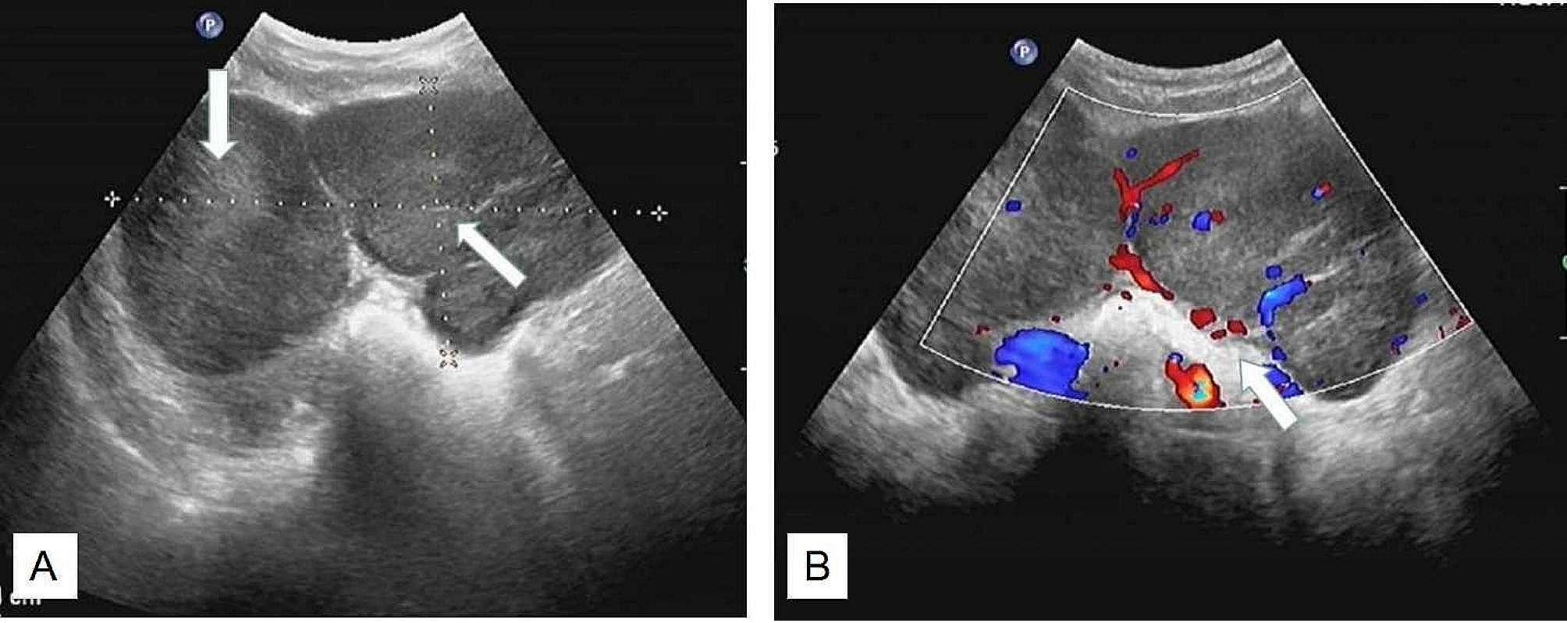
Ultrasound images: (**A**) Ultrasonic probing there are substantial uneven low echo masses sized of 112 mm*58 mm*98 mm (left ovary) and 144 mm*73 mm*83 mm (right ovary) in the pelvic cavity, cling to the uterine corpus, the boundary of the masses are relatively clear. (**B**) the color Doppler shows blood flow signal around the masses (Case 7)

**Fig. 2:**
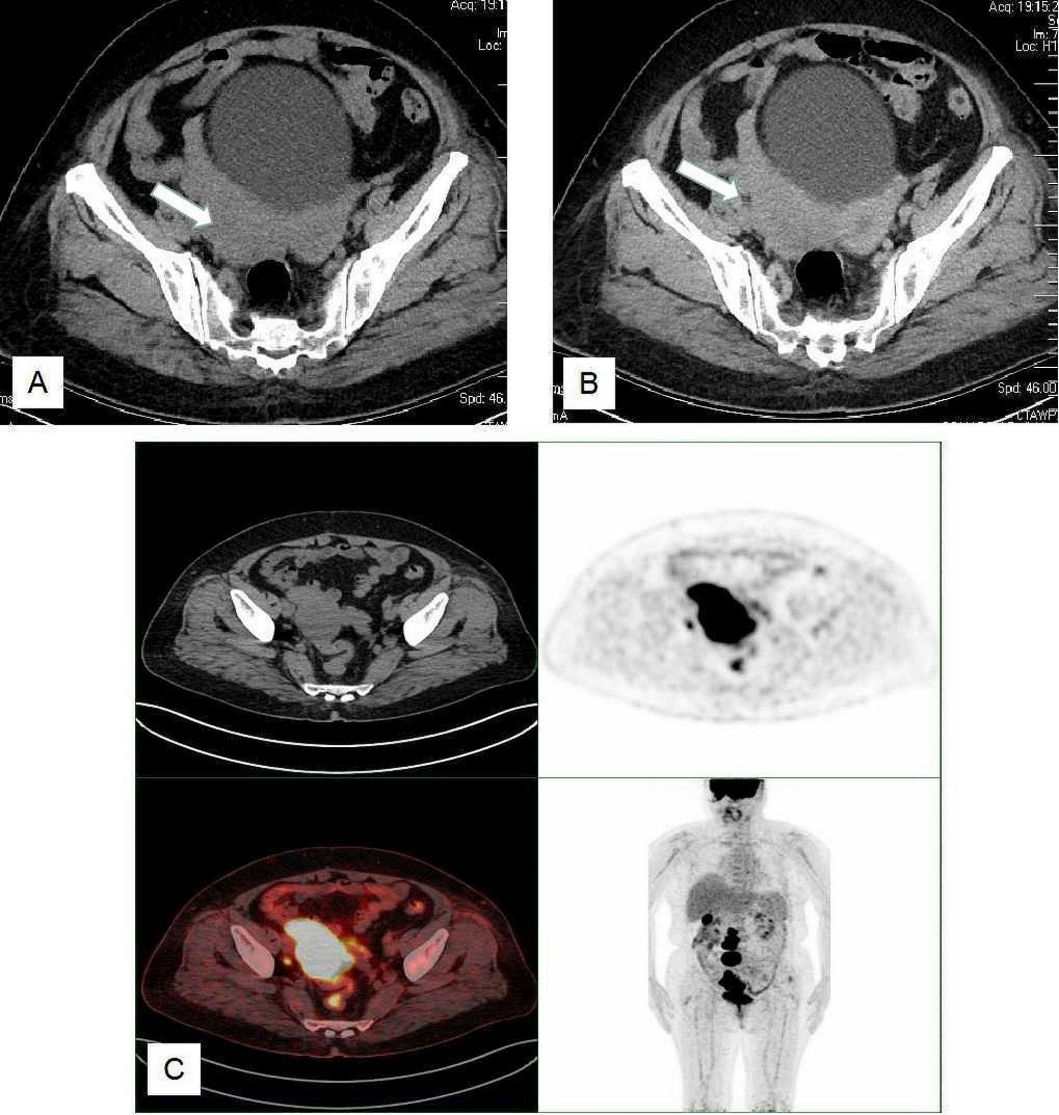
**A.** CT images: The lesion of the right ovary is demonstrated on the axial scan of the CT, and the size is about 45 mm*81 mm. **B.** Axial contrast-enhanced CT images show medium enhancement. **C.** PET-CT images: The PET images of axial view show the uptake in the right ovary lesion and enlarged lymph nodes in the pelvic cavity. Fused images of PET-FDG show the lesion of the right ovary and enlarged lymph nodes with increased uptake. The PET images of coronal view show the uptake in the right ovary lesion and enlarged lymph nodes of the retroperitoneum (Case 11)

**Fig. 3:**
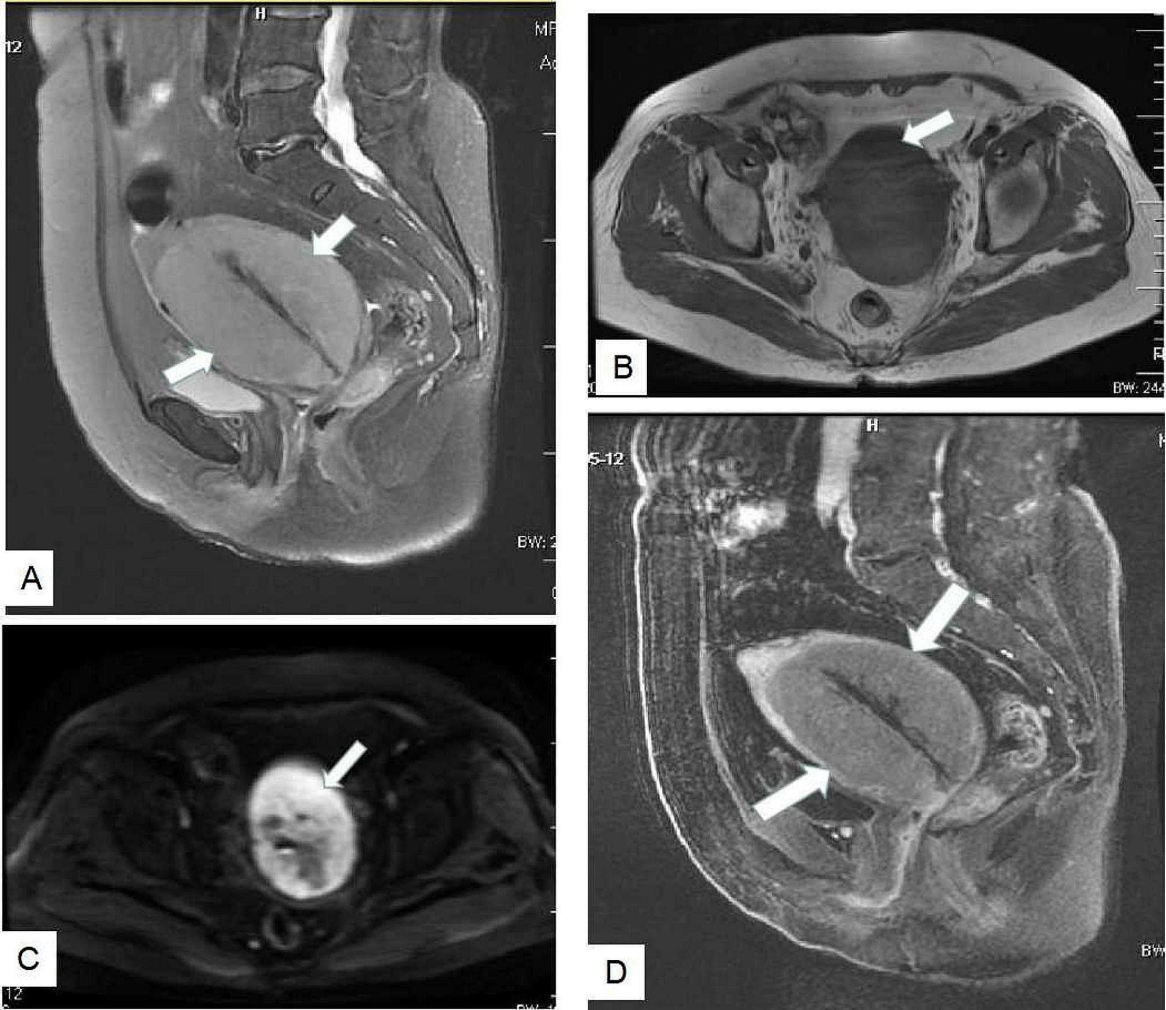
MRI images: (**A**) Sagittal T2-weighted image: enlarged homogeneous mass of uterus shows intermediate intensity. (**B**) Axial T1-weighted image: a large homogenous uterus mass exhibits intermediate signal intensity similar to the muscle. (**C**) Axial DWI image: the uterus mass presents restricted diffusion with high signal intensity. (**D**) Postgadolinium sagittal T1-weighted image: mild homogenous enhancement of the uterus mass after gadolinium administration (Case 9)

Ultrasonography was performed on all patients, and the pelvic masses were hypoechoic solid mass, with the average size of 7.6 cm (3.5-15.0 cm), and color doppler showed mild to moderate blood flow signals within the lesions in 11 patients. Among them, there showed ovaries masses in 8 patients, uterine masses in 5 patients (1 in the uterine muscle layer, 2 in the uterine cavity, 2 in the cervix) (Fig. 1). Eight of 13 patients performed plain and contrast-enhanced CT scan. The pelvic masses indicated irregular shape, with uneven and mainly solid density in plain scan, and mild to moderate enhancement after contrast-enhanced agent injection. Eight cases also had multiple retroperitoneal lymphadenectasis. PET-CT examination was performed in 2 cases and the PET images showed the ovary lesion and enlarged lymph nodes of the retroperitoneum with fludeoxyglucose avid (Fig. 2 ). MRI was performed only in 3 cases and showed large masses in the lower part of uterus and cervical segment (Fig. 3).

### Laboratory examination presentation

The level of serum tumor carbohydrate antigen 125 (CA125) increased in 3 patients before surgery, with an average of 97.6 U/ml (6.7–959.3 U/ml). Serum lactate dehydrogenase (LDH) level increased in 11 cases, with an average of 635.6 U/L (121–2705 U/L) (Table 1). The level of carbohydrate antigen 199 (CA199) was elevated in 1 patient (56.56 U/ml). Serum carcinoembryonic antigen (CEA) and alpha-fetoprotein (AFP) were normal in all patients.

### Treatment and characteristics of pathology

**Fig. 4:**
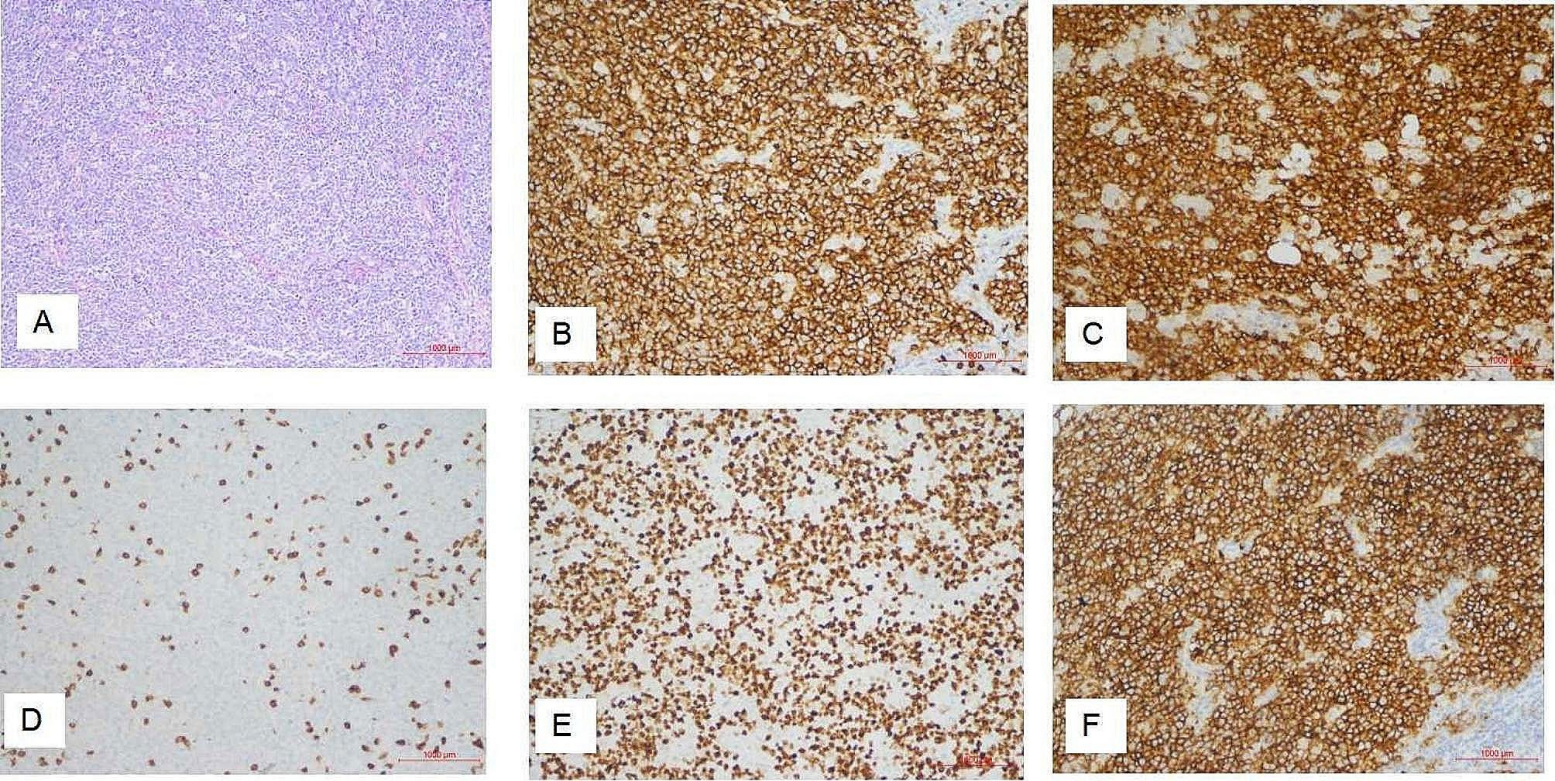
The representation micrographs showing of diffuse large B cell type (DLBCL) extra-nodal lymphoma (hematoxylin-eosin stain for A, ×100; immumohistochemical stain, ×200 for B-F). (**A**) Postoperative specimen of ovary tumor reduction: revealed deep infiltration by sheets of large to medium size cells with oval or round pleomorphic nuclei and scanty cytoplasm. (**B**) CD20 staining: B lymphoid cells population showing CD20 membrane expression is positive. (**C**) CD79a staining: CD79a membrane expression is positive. (**D**) CD3 staining: B lymphoid cells population showing CD3 membrane expression is negative. (**E**) Ki-67 staining: Ki-67 is expressed positivity in 90% tumor cells. (**F**) CD10 staining: CD10 membrane expression is positive

A total of 10 patients underwent surgery, three of them underwent surgical staging for gynecologic malignancies (total hysterectomy, pelvic and para-aortic lymph node dissection, and omentectomy), 2 underwent cytoreductive surgery, 2 underwent total hysterectomy with bilateral adnexectomy, 2 underwent unilateral adnexectomy plus lymphatic biopsy, and 1 underwent radical total hysterectomy, bilateral adnexectomy and pelvic lymph node dissection (Table 1). Diagnostic curettage was performed in 2 cases, and cervical and vaginal puncture biopsy in 1 case. All patients were confirmed as NHL by pathological diagnosis and immunohistochemical examination. Twelve of them were diffuse large B-cell lymphoma and one was follicular lymphoma. Immunophenotypic data showed the expression of one or more pan-B cell antigens (CD10, CD20, CD79a, BCL-2, BCL-6, MUM-1) in all tumors. And negative expression for CD3, CD5, AE1/AE3(-), and cyclin D1 were seen in all cases. Based on the Ann Arbor staging system, 6 cases were classified as stage II and 7 cases were classified as stage IV (Table 2; Fig. 4 ).

**Table 2:** Pathological type and follow-up of 13 patients with PLFGT

Patient number	Pathological type(WHO)	Immunohistochemical results	Clinical staging ( Lymphoma Ann-Arbor)	Chemotherapy	Follow-up (months)
Case1	Diffuse large B-cell lymphoma	CD79α(+), CD20 (+), CD3 (-), CD43:weak(+), MUM-1(+), CD10(-), BCL-6(+), BCL-2(+), CD5(-), PLAP(-), CD99(-),α-inhibin(-), AE1/AE3(-), EMA(-), ki-67: 95%(+), cyclinD1(-)	II	R-CHOP	102 months after surgery, alive with no evidence of recurrence
Case2	Diffuse large B-cell lymphoma	AE1/AE3(-), EMA(-), CD10(-), SMA(-), ER(-), CD20(+), CD79a(+), CD3(-), CD43(-), BCL-2(+/-), MUM-1(+), BCL-6(+), CD5(-), Ki67:90%(+)	II	NK	loss to follow-up
Case3	Diffuse large B-cell lymphoma	AE1/AE3(-), EMA(-), p63(-), CK7(-), LCA(+), CD20(+), CD79a(+), CD3(-), CD43(-), CD10(-), CD5(-), BCL-2(+), BCL-6(+), MUM-1(+), c-Myc:30%(+), CD21(-), CD23(-), cyclinD1(-), Ki-67:90%(+)	II	R-CHOP	98 months after surgery, alive with no evidence of recurrence
Case4	Diffuse large B-cell lymphoma	AE1/AE3(-), EMA(-), CD20(+), CD79a(+), CD3(-),CD43(-), CD10(-), CD5(-), BCL-2(+), BCL-6(+), MUM-1(+), c-Myc:30%(+), CD21(-), cyclinD1(-), Ki-67:80%(+)	IV	R-CHOP	died of acute tumor lysis syndrome 1month after surgery
Case5	Diffuse large B-cell lymphoma	AE1/AE3(-), EMA(-), CD56(-), Syn(-), Des(-), SMA(-), Vim(-), S-100(-), LCA(+), Ki-67:40%(+), CD20(+), CD79a(+), PAX-5(+), CD3(-), CD5(-), CD10(+), BCL-2(+), BCL-6(+), MUM-1(+), CD30(+), cyclinD1(-), MPO(-)	II	R-CHOP	79 months after surgery, recurrence occurred 48 months after surgery
Case6	Diffuse large B-cell lymphoma	AE1/AE(-), CD20(+), CD79a(+), CD3(-), CD43( -), CD5(-), MPO(-) ,BCL-2(+),BCL-6(+), MUM-1(+), CD23(-), CD10(+), CD30(+), cyclinD1(-), Ki-67(+,90%)	IV	refused chemotherapy	Rapidly developed multiorgan failure; died of disease 1 month after surgery
Case7	follicular lymphoma	AE1/AE3 (-), BCL-2(+), BCL-6(+), CD10(+), CD117(-), CD20(+), CD23 displayed follicular dendritic network exists and destruction, CD3(-), CD43(+), CD5(-), CD79(+), c-myc:60%(+), EMA(-), Ki-67:80%(+), PLAP(-), Inhibin-a(-), MUM-1(+), cyclinD1(-), CISH:EBER(-)	IV	R-CHOP	38 months after surgery, alive with no evidence of recurrence
Case8	Diffuse large B-cell lymphoma	AE1/AE3(-), EMA(-), CD20(+), PAX-5(+), CD3(-), CD43(-), CD5(-), CD10(+), BCL-2(+), BCL-6(+), MUM-1(+), CD79a(+), C-myc:50%(+), CD30(-), CD21(-), TdT(-), cyclinD1(-), Ki-67:90%(+)	II	R-CDOP	33 months after surgery, alive with no evidence of recurrence
Case9	Diffuse large B-cell lymphoma	AE1/AE3(-), EMA(-), CD20(+), PAX-5(+), CD3(-), CD43(-), CD5(-), CD10(+), CD79a(+), BCL-2(+), BCL-6(+), MUM-1(+), C-myc:50%(+), CD30(-), CD21(-), cyclinD1(-), Ki-67:70%(+), CISH:EBER(-)	IV	R-CHOP	32 months after surgery, alive with no evidence of recurrence
Case10	Diffuse large B-cell lymphoma	AE1/AE3(-), EMA(-), CD20(+), CD3(-), CD43(-), BCL-2(+), BCL-6(+), CD10(+), CD79a(+), CD15(-), CD21(-), CD30(-), CD5(-), CD99(-), c-myc:40%(+), Inhibin-a(-), MPO(-), MUM-1(+), Ki-67:70%(+), cyclinD1(-), CISH:EBER(-)	IV	R-CHOP	31 months after surgery, alive with no evidence of recurrence
Case11	Diffuse large B-cell lymphoma	AE1/AE3(-), EMA(-), CD20(+), PAX-5(+), CD79a(+), CD3(-), CD43(-), BCL-2(+), BCL-6(+), CD10(+), CD15(-), CD21:(+), CD30(-),CD5(-),, c-myc:50%(+), Inhibin-a(-), MUM-1(+), cyclinD1(-), Ki-67:90%(+), CISH:EBER(-)	IV	R-CHOP	22 months after surgery, alive with no evidence of recurrence
Case12	Diffuse large B-cell lymphoma	AE1/AE3(-), EMA(-), CD20(+), PAX-5(+), CD3(-), CD43(-), CD21(-), BCL-2(+), BCL-6(+), CD10(+), CD79a(+), MUM-1(+), CD5(-), c-myc(+ 80%), CD30(-), cyclinD1(-), ki-67(+ 80%), CISH:EBER(-)	IV	refused chemotherapy	died of COVID-19 3 month after surgery
Case13	Diffuse large B-cell lymphoma	AE1/AE3(-), EMA(-), p63(-), CK7(-), LCA(+), CD20(+), CD79a(+), CD3(-), CD43(-), CD10(+), CD5(-), BCL-2(+), BCL-6(+), MUM-1(+), c-Myc:30%(+), CD21(-), CD23(-), cyclinD1(-), Ki-67:90%(+)	II	R-CHOP	9 months after surgery, alive with no evidence of recurrence

PLFGT Primary lymphoma of the female genital tract

Ten patients received chemotherapy in hematology department after surgery. Nine patients were treated with R-CHOP (rituximab, cyclophosphamide, doxorubicin, vindesine, and prednisone) chemotherapy. A 70-year-old patient with hypertension and coronary heart disease was treated with R-CDOP (rituximab, cyclophosphamide, doxorubicin liposome, vinodicine, and meprednisolone). Two patients refused to receive chemotherapy after surgery, and one patient was lost to follow-up.

### Prognosis

The follow-up of patients ended in Aug. 2023, and the average observation time was 31 months (range, 0-102 months). At the time of last follow up, 8 patients were alive with no evidence of disease, 1 had recurrent disease, 3 died (one died of multiorgan failure, one acute tumor lysis syndrome, and one COVID-19), and 1 was lost to follow-up. The median survival time was 32 months (range, 1-102 months).

## Discussion

PLFGT is a rare occurrence. The true origin of PLFGT remains unclear. Bhagat et al. [10] and Au et al. [11] found that the most common sites of PLFGT were cervix and ovary, while vagina and uterus were scarce. Stabile et al. [8] and Slonimsky et al. [7] reported that ovary was the most common site of PLFGT, followed by cervix. In our study, we observed that the ovary (8/13, 61.5%) was the most common site of PLFGT, followed by the uterus (3/13, 23.1%) and cervix (2/13, 15.4%). In line with previous research, the ovary remains the most prevalent site of PLFGT. As mentioned in previous studies [7, 8, 10, 11], diffuse large B cell lymphoma (DLBCL) is the most common histological type. These findings were also consistent with our study, most tumors were of DLBCL histology (12/13, 92.3%), while follicular subtype lymphoma was relatively rare observed (1/13, 7.7%).

However, it’s a challenge to distinguish between primary and secondary ovarian lymphoma, especially in cases with both nodal and extranodal involvement. Fox et al. [12] had proposed the following diagnostic criteria for primary ovarian lymphoma: (1) tumor confined to ovarian regional lymph nodes or adjunctive organs at diagnosis, (2) absence of abnormal cells in the peripheral blood or bone marrow, and (3) any extraovarian disease occurred within a few months following the appearance of the ovarian lesion. These criteria have been further employed to diagnose primary lymphoma of the female genital tract arising at other sites such as the uterus [13]. According to the diagnostic criteria put forward by previous studies, all patients in our study met the diagnosis of PLFGT.

Previous literature had reported that the onset age of PLFGT is a long span, (range, 20–80 years), and the median age is older (range, 44–54 years old) [8, 14]. In this study, the onset age ranged from 30 to 78 years old, with a median age of 67. The age span was basically similar to previous studies, but the median age was older, which may result from a limited research sample. Most patients in this study were postmenopausal patients, whose age of onset was similar to that of female reproductive tract malignancies such as ovarian cancer and endometrial cancer. The symptoms of PLFGT are lack of speciality and similar to those of cervical, endometrial or ovarian cancer, including abnormal vaginal bleeding, pelvic mass, abdominal distension, abdominal pain and ascites. The analysis of clinical data from 13 cases in this study revealed that vaginal bleeding was a common presentation of uterine or cervical lymphoma, while pelvic mass, abdominal pain, and distension were predominant manifestations of ovarian lymphoma. These findings aligned with the report of Frey et al. [15]. Due to the similarities in initial symptoms and age of onset between PLFGT and gynecological malignancies, they are frequently misdiagnosed as malignancies of the female reproductive tract prior to surgery. In contrast to nodal NHL, which typically exhibits prodromal symptoms such as fever, night sweats, weight loss, or ‘B’ symptoms, patients with PLFGT rarely present with these symptoms [10, 16, 17]. Our research also revealed that none of 13 patients exhibited these symptoms. Consequently, distinguishing PLFGT from malignancies in the female genital tract becomes more challenging.

As a routine examination item for lymphoma, the serum LDH elevated in 31% of extranodal lymphomas patients and was a predictor of poor prognosis [18]. By analyzing the preoperative serum LDH of 11 patients with primary female genital lymphoma, Shen et al. [19] considered that serum LDH might be a tumor marker of PLFGT, which could aid in distinguishing PLFGT from gynecological malignancy. In our study, the increase of serum LDH accounted for 84.6% (11/13). So preoperative elevated serum LDH can be applied as a potential tumor marker for PLFGT, although the result still requires further investigation. PLFGT patients are usually diagnosed in gynecology department first. And as gynecologists, we always focus on the serum levels of CA125, CA199, epididymal protein 4 (HE4), CEA, and AFP. Previous studies had also shown that patients with PLFGT can be accompanied by an elevation of serum CA125, and serial serum CA 125 measurements are useful for monitoring patient response to therapy [10, 15, 20, 21]. However, in our study, only 23.1% (3/13) of the PLFGT patients had elevated serum CA125 before surgery. So we consider serum CA125 could be elevated in some PLFGT patients, but whether CA125 is related to PLFGT requires further study of large sample cases.

Given the lack of typical clinical findings in PFGLT patients, radiological or imaging studies have also been evaluated as useful diagnostic method [14, 22]. Previous studies [7, 23] showed that the main ultrasonographic manifestations of PLFGT were hypoechoic or extremely hypoechoic solid lesions that were relatively large in size (86.7% is greater than 5 cm), with clear boundaries and regular shapes, and ascites is rare. The presence of large, bilateral, homogeneous ovarian masses is a clue to the diagnosis of ovarian lymphoma. Abdominal and pelvic CT can help identify the involvement sites of disease [24]. PET-CT is also useful to track disease regression/progression and recurrence [25, 26]. Marin et al. [27] demonstrated there are several imaging findings can help to differentiate PLFGT from other malignancies. In our study, imaging examinations of all patients showed hypoechoic solid masses in the adnexal region/uterus, and most of them were large lesions, with an average size of 7.6 cm (3.5-15.0 cm), and ascites were rare (only 1 case). CT examinations showed not only large lesions in uterus or ovary, but also multiple retroperitoneal lymph node enlargement. So we consider that if patients with adnexal region/uterine mass with a size greater than 5 cm, accompanied by multiple lymph node enlargement in retroperitoneum without ascites, the possibility of PLFGT should be considered before surgery.

It is not easy to differentiate PLFGT from female genital tract primary disease even if using frozen section. Vang et al. [28] stated that the definitive diagnosis between poorly differentiated carcinoma and NHL can be difficult using frozen section only, particularly for pathologists who do not specialize in deal with lymphoma. And the final definitive diagnosis still relies on other methods such as flow cytometry, cytogenetics, immunohistochemistry, and molecular diagnostic. In our study, the intraoperative frozen sections were done in all eight patients with ovarian masses. But only two cases were diagnosed as lymphoma at the time of frozen section. Other six cases were misdiagnosed as undifferentiated carcinoma, epithelial tumor, or granulosa cell tumor, so they underwent staging surgery or debulking surgery for ovarian cancer. However, in our study, three patients were pathological diagnosed as DLBLC after cervical and endometrial biopsy. The diagnosis of PLFGT by biopsy has not been extensively discussed in previous studies. Kasai et al. [29] reported two patients with clinical manifestations of pelvic masses were performed puncture biopsy, which were confirmed to be NHL by pathological diagnosis, and were completely relieved after R-CHOP chemotherapy. Their study suggested that needle biopsy is a reliable way to diagnose PLFGT. However, due to the quantity of biopsy tissue, sometimes this examination lacks certain accuracy. Yadav et al. [30] reported that a 14-year-old female was performed puncture biopsy, due to lack of sufficient tissue, immune cytochemistry was unable to perform, and finally the patient underwent exploratory laparotomy to remove the bilateral ovarian masses and Burkitt’s lymphoma was confirmed by histopathological examination. Imaging-guided biopsy is feasible to patients with pelvic mass and suspicious PLFGT. But diagnostic methods (such as flow cytometry, cytogenetics, immunohistochemistry, and molecular diagnostic, etc.) are unable to perform in some cases due to the insufficient biopsy tissue. So surgery is still required to obtain a definitive diagnosis for these patients.

Due to the rare incidence of PLFGT, there are currently no randomized clinical trials or specific guidelines pertaining to the treatment. It was reported in previous literatures that most PLFGT patients underwent tumor-related surgery, and after pathological diagnosis, combination chemotherapy with R-CHOP was initiated [3, 4]. The decision to administer radiotherapy based on the histological subtype of the tumor, disease extension, and patient-related factors [17, 31–33]. In our study, 10 patients received chemotherapy, 9 received R-CHOP regimen with the median survival time of 38 months (ranging from 1 to 102 months), and 1 received R-CDOP regimen with survival time of 33 months. Some researchers [34, 35] reported that compared with R-CHOP regimen, R-CDOP regimen carried lower risks of adverse effects and non-serious cardiovascular events, and its efficacy was basically the same as that of R-CHOP regimen. As in our study, one patient who received the R-CDOP regimen, which may be related to the complication of hypertension and CHD complications. Currently, the main chemotherapy regimen for PLFGT is still R-CHOP regimen, and R-CDOP chemotherapy can be used in patients at risk of heart disease. However, due to the few number of sample and short follow-up period in this study, which treatment method for PLFGT patients will benefit the most is still needed further research to confirm.

## Conclusions

In conclusion, PLFGT is a rare extranodal lymphoma, usually presents as gynaecological-related symptoms, and is often misdiagnosed as a gynecological malignancy due to the lack of specific clinical examination methods. However, patients with PLFGT still have some certain features, such as a large pelvic solid mass, multiple enlarged retroperitoneal lymph nodes, rare ascites and concurrent elevation of serum LDH levels. Therefore, suspicious lymphoma should be considered when the patient presents these signs. And image guided biopsy can be performed to obtain definitive diagnosis. But surgery is still required if the biopsy has no definite result. This study has some limitations. Our study design was a retrospective chart review of a relatively small study group, with consequent limitations of data accessibility. Although early diagnosis is difficult to establish,it is essential to develop an appropriate personalized treatment plan. We suggest conducting larger multi-institutional studies registry to assist in elucidating the optimal management of women with PLFGT.

## Data Availability

The datasets used during the current study are available from the corresponding author on reasonable request.

## Abbreviations

PLFGT: Primary lymphoma of the female genital tract
LDH: Lactate dehydrogenase
CA125: Carbohydrate antigen
NHL: Primary non-Hodgkin lymphoma
R-CHOP: Cyclophosphamide, hydroxydaunorubicin, oncovin, and prednisone with rituximab
R-CDOP: Rituximab, cyclophosphamide, doxorubicin liposome, vinodicine, and meprednisolone
WHO: World Health Organization
CHD: Coronary heart disease
DLBCL: Diffuse large B-cell lymphoma
